# Determinants of CD4 count recovery among severely immunosuppressed HIV patients initiated on antiretroviral therapy: a prospective cohort study in KwaZulu-Natal, South Africa

**DOI:** 10.3389/fpubh.2026.1766369

**Published:** 2026-03-04

**Authors:** Chiedza Elvina Mashiri, Retius Chifurira, Knowledge Chinhamu, Jesca Mercy Batidzirai

**Affiliations:** 1 Department of Applied Mathematics and Statistics, Midlands State University, Gweru, Zimbabwe; 2 Department of Statistics, School of Agriculture and Science, University of KwaZulu-Natal, Durban, South Africa; 3 Department of Statistics, School of Agriculture and Science, University of KwaZulu-Natal, Pietermaritzburg, South Africa

**Keywords:** art, CD4 count, recovery time, risk factors, survival models

## Abstract

**Background:**

For people living with HIV (PLWH), CD4 count serves as an effective indicator of response to antiretroviral therapy (ART) and a predictor of morbidity and mortality before and after ART initiation. In PLWH on ART, changes in CD4 count reflect immunological response to treatment. CD4 count recovery after ART initiation is defined as achieving a CD4 cell count of ≥500 cells/mm^3^. This study utilised survival analysis techniques to determine time to CD4 count recovery and to identify determinants among PLWH with severe immunosuppression who were initiated on ART in KwaZulu-Natal, South Africa.

**Materials and methods:**

Secondary data were collected by CAPRISA from June 2004 to August 2013, with monthly hospital visits. Time to CD4 recovery was calculated from ART initiation to the first CD4 count ≥500 cells/mm^3^. Participants were followed until recovery; those who did not recover, were lost to follow-up, or died were censored. Kaplan–Meier curves estimated median survival time, while Cox and Weibull regression models identified risk factors associated with CD4 recovery.

**Results:**

Among 2,528 participants (median age 32 years), 1,803 had viral load <400 copies/mL, 1,589 were females, and 524 had TB. By the end of the study, 727 achieved CD4 recovery. Kaplan–Meier estimates showed median time to recovery of nearly 40 months for females and 59 months for males. The Weibull model outperformed the Cox model. Male participants had a 41% lower hazard of recovery than females [HR: 0.589, 95% CI: 0.497–0.698], while rural care was associated with a 49% higher hazard of recovery than urban settings [HR: 1.494, 95% CI: 1.228–1.818]. TB-coinfected participants had a 39.5% lower hazard [HR: 0.605, 95% CI: 0.489–0.748], whereas those with viral suppression had a 49% higher hazard of recovery [HR: 1.489, 95% CI: 1.213–1.826].

**Conclusion:**

Delayed CD4 recovery among males, urban residents, TB-coinfected participants, and those with unsuppressed viral load (>400 copies/mL) underscores the need for targeted, differentiated HIV care strategies to accelerate immunological recovery and reduce HIV-related morbidity. Policymakers should prioritise male-focused interventions, strengthened TB–HIV integrated services, intensified adherence and viral load monitoring, and context-specific interventions in urban settings to accelerate immunological recovery and reduce HIV-related morbidity.

## 1 Introduction

Human Immunodeficiency Virus/Acquired Immunodeficiency Syndrome (HIV/AIDS) is a disease deeply intertwined with social and economic inequalities in many developing nations, including South Africa, where it disproportionately impacts individuals in lower socioeconomic strata and impoverished communities (1). Since 2007, the South African government has made significant strides toward ending its AIDS epidemic by 2030, showcasing that combining scientific evidence with political commitment can save lives (2). The country continues to roll out programs that support various HIV and AIDS prevention strategies, including medical male circumcision, prevention of mother-to-child transmission, and accessible antiretroviral treatment (ART), which is provided free of charge to anyone who tests positive, irrespective of their CD4 count. These subsidised initiatives offer a cost-effective solution for the government to meet its public health goals.

KwaZulu-Natal (KZN), the most populous province in South Africa, has the highest HIV prevalence, with 27.6% of adults living with the virus (3, 4). Following the introduction of ART, there has been a decline in mortality, morbidity, and HIV transmission among those living with HIV (5–7). The need for preventative treatment against opportunistic infections is guided by the CD4 count, a key indicator of a patient’s immune status (8). The recovery of CD4 cell counts after starting ART is a promising predictor of clinical outcomes for HIV patients, correlating with a healthier immune system and enhanced life expectancy (9).

During the first 3 months of suppressive antiretroviral therapy (ART), the rate of CD4 cell recovery is low but progressively improves over time (10). If viral suppression through ART is sustained, most individuals will eventually achieve normal CD4 counts (≥500 cells/mm^3^). However, about 15–20% of those who start ART with very low CD4 counts (around 200 cells/mm^3^) may continue to experience persistently low CD4 levels (11). This clinical immunological failure to respond to ART is linked to factors such as age, sex, co-infection with viral hepatitis, initial CD4 + T-cell counts, prolonged untreated HIV infection, and heightened morbidity and mortality (12–14). The inability to recover CD4 counts is associated with an elevated risk of non-AIDS-related health issues and mortality, including cardiovascular diseases, pneumonia, osteoporosis and fractures, liver disease, and cancers related to infections (15–17).

Recent studies have used various statistical methods to assess HIV in KwaZulu-Natal. For example, Mchunu et al. (18) used joint modelling of time-to-event data to assess CD4 cell count and mortality among patients who initiated antiretroviral therapy. The analysis showed that CD4 count can be utilised as a measure of immunological failure and is associated with mortality. Dawood et al. (19) used Kaplan–Meier and multivariable proportional risk models to examine factors associated with hepatitis B prevalence and persistence. The authors concluded that the provision of HIV care and treatment in high Hepatitis B Virus (HBV)-burden settings provides a missed opportunity for HBV screening, immunisation and care provision. Another study (20) utilised Kaplan–Meier curves, log-rank tests, and multivariate proportional hazards models to focus on mortality and treatment response in patients aged 50 years and older. The results showed that older HIV-infected patients were associated with higher mortality compared to the younger cohort. Older patients have a risk of developing diabetes and hypertension. Naidoo et al. (21) used Kaplan–Meier and multivariable proportional hazards models to analyse factors associated with male mortality. Results showed men had more advanced HIV disease and a higher mortality rate compared to women. Although the findings from these studies remain valid and relevant, population dynamics and other health indicators may have changed in recent years.

Despite the introduction of universal test and treat (UTT) in South Africa in 2016, a substantial proportion of people living with HIV (PLWH) continue to initiate antiretroviral therapy (ART) at CD4 counts ≤200 cells/mm^3^, underscoring persistent challenges in early HIV diagnosis and timely linkage to care (22, 23). This pattern highlights a critical disconnect between ART initiation and effective immunological recovery, warranting empirical investigation. Although CD4 count recovery following ART initiation has been extensively studied, evidence remains limited for individuals who begin treatment with advanced immunosuppression, particularly in high-burden, resource-constrained settings. To address this gap, this study applies survival analysis methods to estimate the time to CD4 count recovery and to identify its key determinants among PLWH receiving ART in KwaZulu-Natal, South Africa. The findings are expected to inform resource allocation, clinical management, and policy formulation to improve treatment outcomes.

## 2 Materials and methods

### 2.1 Data

This study utilised data from the South African Centre for the AIDS Program of Research (CAPRISA), which enrolled 4,013 patients to initiate antiretroviral therapy between June 2004 and August 2013. Out of these, 2,528 patients met the inclusion criteria, defined as having a CD4 count below 200 cells/mm^3^ or a WHO stage 4 AIDS-defining illness. Baseline data recorded included CD4 counts, viral load, demographic information, and medication regimens. CD4 counts and viral loads were measured at the start of the study, every 6 months, or as needed for clinical reasons. The research was conducted in two locations: eThekwini, an urban area, and Vulindlela, a rural one. Patients who missed three or more consecutive visits and could not be physically tracked were classified as lost to follow-up.

### 2.2 Ethical considerations

Approval for data usage and analysis was granted by the Biomedical Research Ethics Committee at the University of KwaZulu-Natal (Ref: E248/05). Additionally, ethical clearance was obtained through CAPRISA.

### 2.3 Statistical analysis

The study aimed to determine the time to CD4 recovery in patients living with HIV after initiating antiretroviral therapy (ART). Descriptive statistics of demographic characteristics were categorized based on whether patients achieved a CD4 count of ≥500 cells/mm^3^ or <500 cells/mm^3^. The *t*-test and chi-squared tests were used to assess the association between CD4 count and other variables; *p*-values < 0.05 indicated statistical significance. The primary event of interest was reaching a CD4 count of 500 cells/mm^3^ or higher.

Survival differences among various covariates were assessed using Kaplan–Meier curves and the log-rank test. Additionally, a Cox proportional hazards model was utilised to simultaneously evaluate factors associated with CD4 count recovery.

Both univariate and multivariate survival analyses were conducted on baseline characteristics, with the multivariate analyses focusing on significant variables from the univariate analysis with *p*-values <0.05. The Cox model was adjusted for baseline covariates, including age, gender, tuberculosis prevalence, study site, viral load, and baseline CD4 count. The assumptions of the Cox proportional hazards model were evaluated using the Global Schoenfeld test; a *p*-value <0.05 indicated non-proportionality, leading to the adoption of the Weibull parametric regression model, which accommodates a non-constant baseline hazard that can increase or decrease monotonically.

Results from the Weibull model indicated that age, tuberculosis prevalence, location, viral load, gender, and baseline CD4 count were statistically significant. Statistical analysis was performed using the R surv package version 4.1.2.

## 3 Results

### 3.1 Exploratory data analysis for baseline characteristics

Table 1 presents the baseline characteristics of the 2,528 people living with HIV who initiated antiretroviral therapy (ART) with a baseline CD4 count <200 cells/mm^3^ in accordance with the 2004 eligibility criteria (24). Overall, 727 participants (29%) achieved CD4-count recovery, defined as attaining a CD4 count ≥500 cells/mm^3^ during follow-up. Among those who achieved CD4 recovery, 542 (74.5%) were female, and 557 (76.6%) resided in rural areas. The median age was similar between participants who achieved CD4 recovery and those who did not (median 32 years in both groups), although the difference was statistically significant (*p* < 0.001). Baseline clinical characteristics differed between the two groups. Participants who achieved CD4 recovery were more likely to present with earlier WHO clinical stages, including stage 1 (*n* = 105) and stage 2 (*n* = 136), compared with those who did not recover (*p* = 0.03). The majority of participants who achieved CD4 recovery did not have tuberculosis at baseline (*n* = 594), and a smaller proportion had a baseline viral load >400 copies/mL (*n* = 113), with viral load suppression being significantly associated with CD4 recovery (*p* < 0.001). The median baseline CD4 count among participants who achieved CD4 recovery was 109 cells/mm^3^ (IQR: 52–154), which was significantly higher than among those who did not recover (*p* < 0.001).

**Table 1:** Baseline characteristics by CD4 count of 500 cells/mm^3^.

Baseline characteristics	CD4 < 500 (*N* = 1801)	CD4 ≥ 500 (*N*=727)	*p*-value
Site, *n* (%)			
eThekwini (urban)	893 (35%)	170 (7%)	<0.001
Vulindlela (rural)	908 (36%)	557 (22%)	
Age*, median (IQR)	32 (29–39)	32 (28–38)	<0.001
WHO Stage**n (%)			
1	216 (8%)	105 (4%)	0.03
2	334 (13%)	136 (5%)	
3	1,024 (41%)	419 (17%)	
4	221 (9%)	65 (3%)	
TB status, *n* (%)			
TB present	391 (15%)	133 (5%)	0.06
No TB	1,410 (56%)	594 (24%)	
Viral load (copies/mL)			
> 400	612 (24%)	113 (5%)	<0.001
≤ 400	1,189 (47%)	614 (24%)	
Regimen, *n*			
Regimen 1 (first line)	1,448	586	0.95
Gender, *n* (%)			
Female	1,047 (41%)	542 (22%)	<0.001
Male	754 (30%)	185 (7%)	
CD4 count median (IQR)	88 (42–141)	109 (52–154)	<0.001

*3 missing age, **8 missing WHO stages.

### 3.2 Kaplan–Meier curves

**Figure 1:**
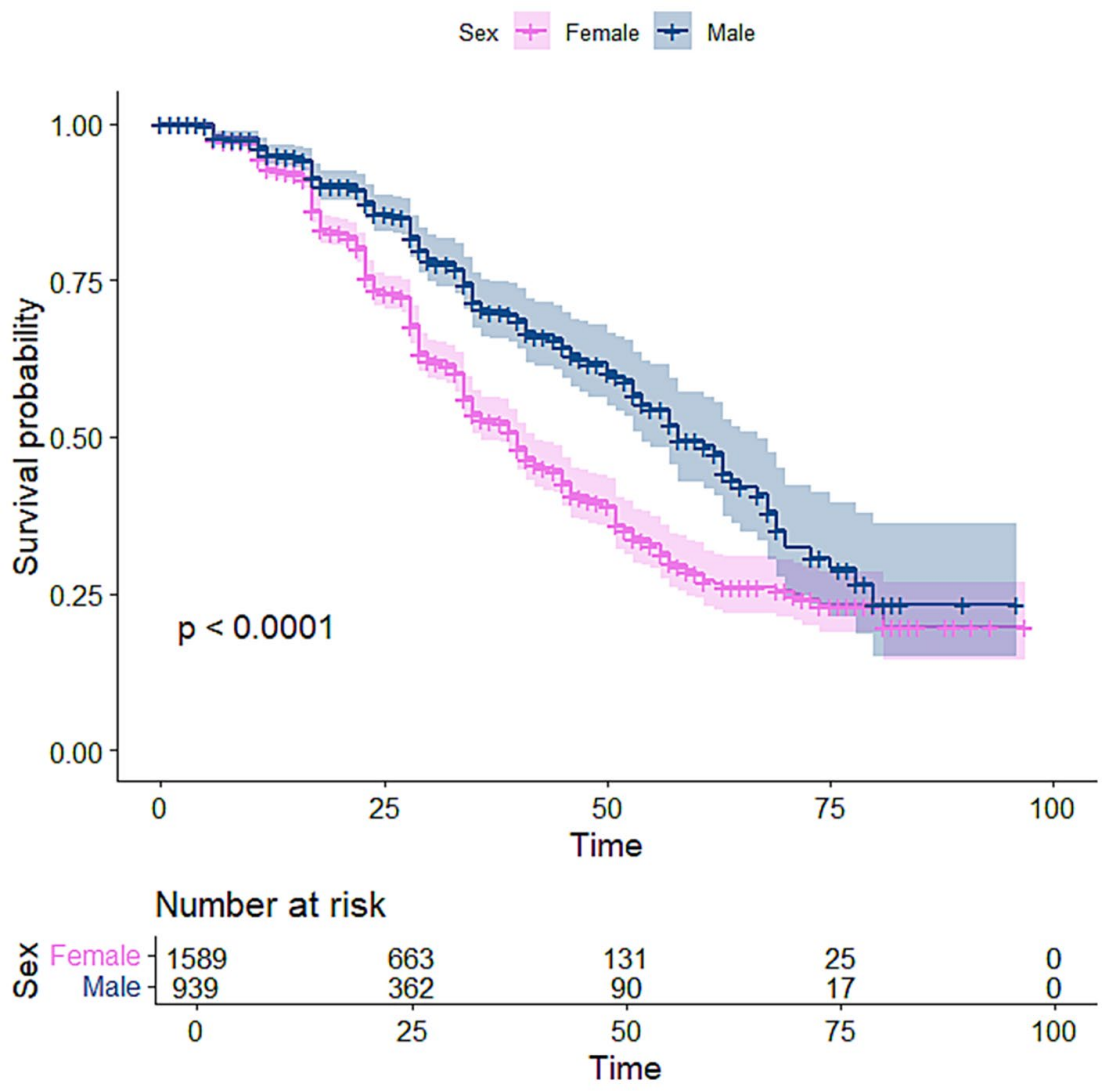
Kaplan–Meier curve for gender.

**Figure 2:**
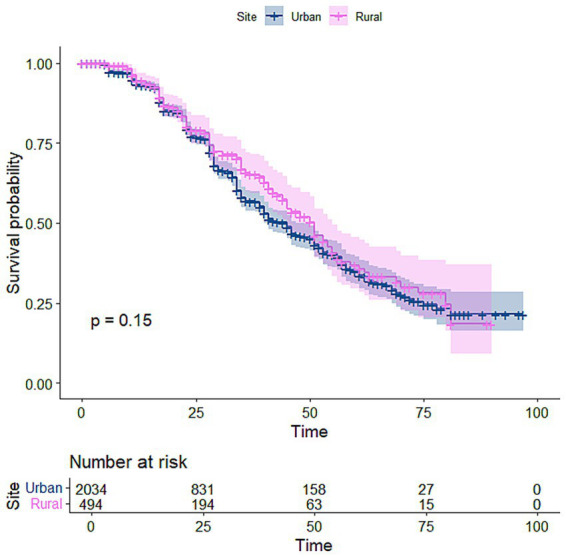
Kaplan–Meier curve for location.

**Figure 3:**
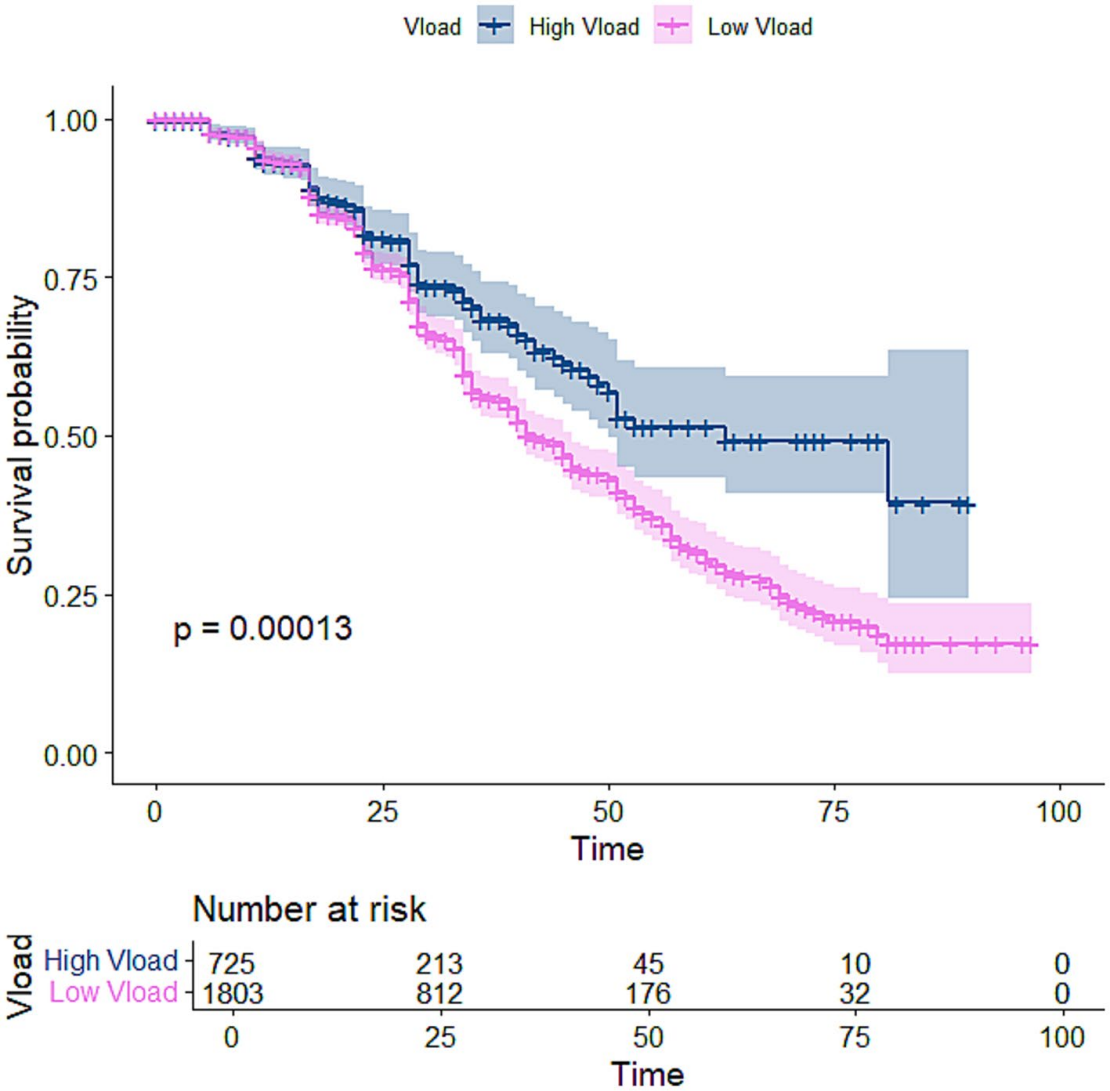
Kaplan–Meier curve for viral load.

**Figure 4:**
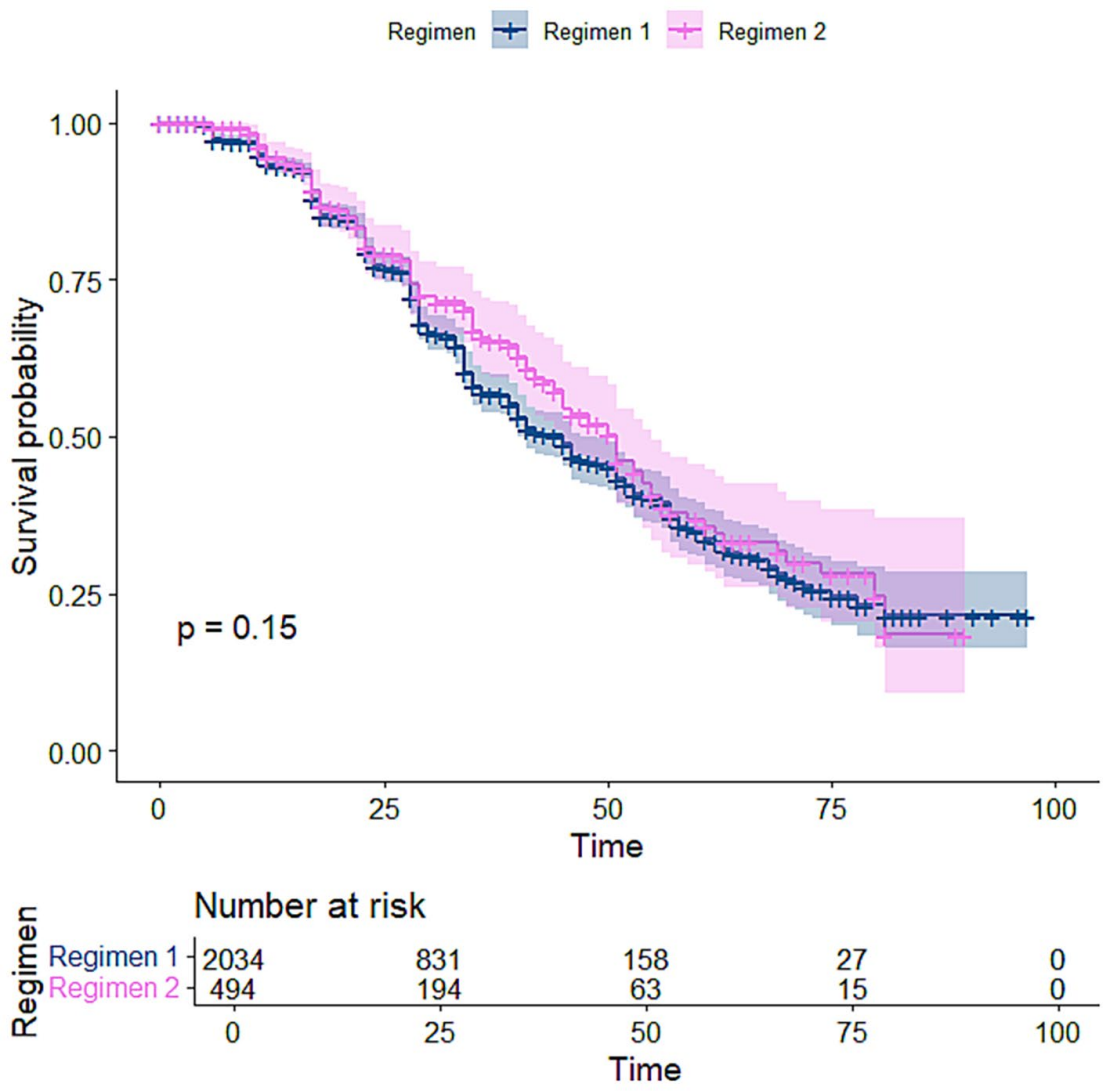
Kaplan–Meier curve for regimen.

The Kaplan–Meier (K-M) curve visualises and estimates survival rates over time, showing the probability of an event. Figure 1 suggests that males recovered at a higher rate than females. Figure 2 shows that patients from rural areas recovered at a lower rate than those from urban areas. The Kaplan–Meier curve in Figure 3 indicates that patients with high viral load had a higher chance of CD4 count recovery than those with low viral load. Figure 4 shows a higher rate of recovery in patients on first-line ART compared to those on second-line ART.

### 3.3 Cox proportional hazard model

Table 2 displays the maximum likelihood estimates of the covariates derived from both the univariate and multivariate Cox proportional hazards models. The significant variables identified in the univariate analysis were used to construct the multivariate model. The results indicated that age, tuberculosis, location, viral load, gender, and baseline CD4 count were statistically significant predictors of CD4 recovery.

**Table 2:** Cox proportional hazard.

Characteristics	Univariate Cox model	
	HR (95% CI)	*p*-value
Gender (Ref: female)*** Male	0.584 (0.494–0.690)	<0.001
Age***	0.980 (0.971–0.990)	<0.001
TB (Ref: no TB present)*** TB	0.759 (0.628–0.918)	0.004
WHO stage1	0.366 (0.090–1.484)	0.159
WHO stage2	0.314 (0.078–1.272)	0.105
WHO stage3	0.372 (0.093–1.494)	0.141
WHO stage4 (Ref)		
Site (Ref: urban)*** Rural	1.307 (1.099–1.555)	0.002
Regimen (Ref: first line treatment) Second line treatment	0.871 (0.724–1.047)	0.141
Viral load (Ref: >400copies/mL) ≤400copies/mL***	1.467 (1.200–1.793)	<0.001
Baseline CD4 count***	1.004 (1.003–1.006)	<0.001

***Indicates significant variables.

Notably, the hazard of recovery decreased with advancing age, with a hazard ratio (HR) of 0.980 (95% CI: 0.971–0.990). Males had a significantly lower likelihood of recovery compared to females, with an HR of 0.584 (95% CI: 0.494–0.690). This means that, at any given time after ART initiation, males were significantly less likely to achieve CD4 recovery than females. Patients in rural areas had a 30.7% [HR: 1.307 (95% CI: 1.099–1.555)] higher hazard of CD4 recovery compared to urban patients. This means rural patients achieved CD4 recovery at a faster rate than urban patients did. Patients with viral loads of 400 copies/mL or lower were 1.5 times more likely to recover their CD4 counts compared to those with viral loads exceeding 400 copies/mL, with an HR of 1.489 (95% CI: 1.213–1.826). This indicated that viral suppression is strongly associated with faster immune recovery. While the WHO stage and treatment regimen did not reach statistical significance, they remain crucial considerations. For patients on first-line treatment, achieving a CD4 count of 500 cells/mm^3^ is expected in comparison to those receiving second-line therapy.

### 3.4 Schoenfeld residuals

**Figure 5:**
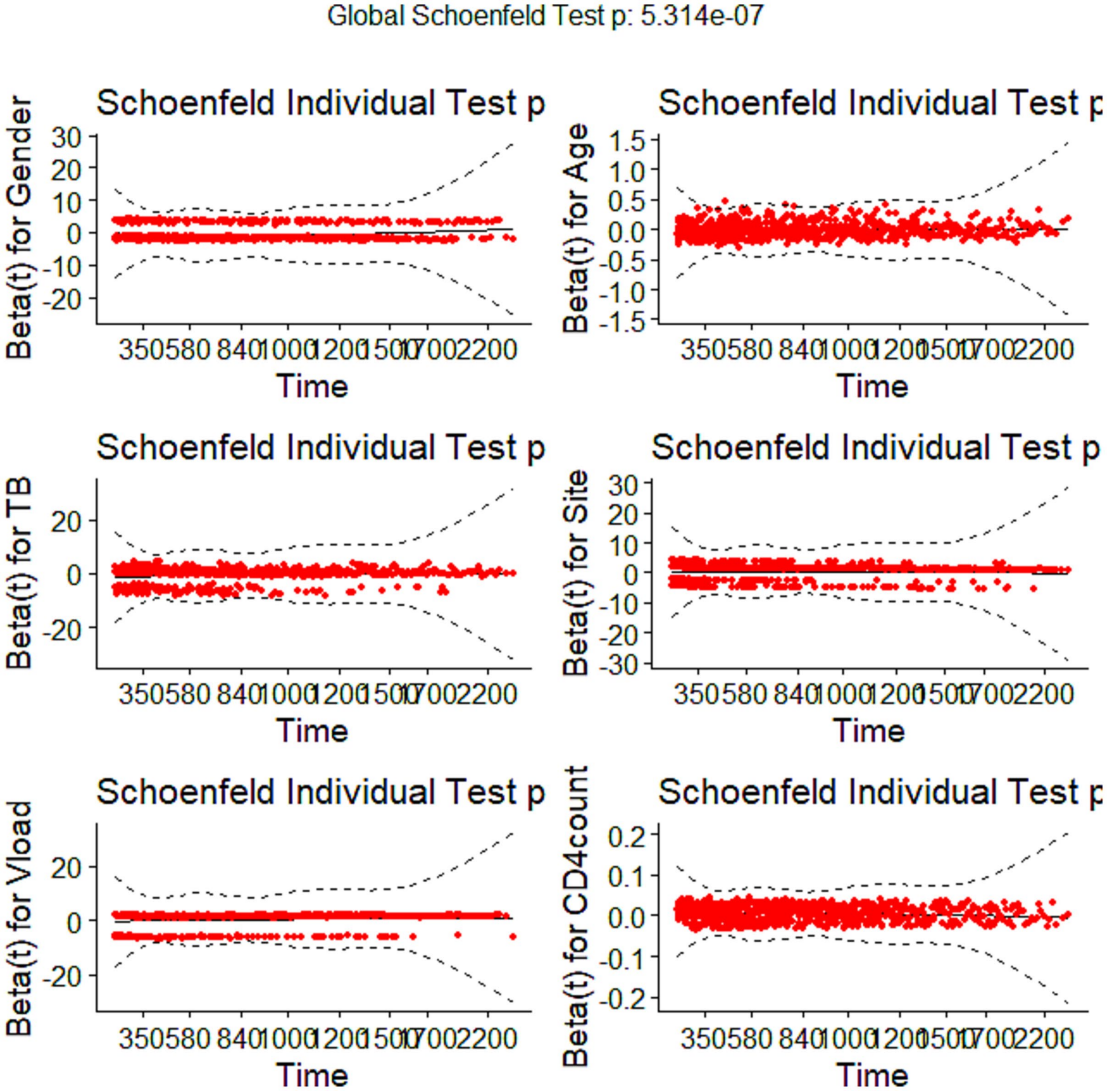
Schoenfeld residual.

Figure 5 shows Schoenfeld residuals, which are a standard measure for Cox Proportional hazard assumptions. A low *p*-value <0.05 suggests that the proportional hazards assumption is violated for that covariate. The results in Figure 5 showed that gender (*p*-value = 0.0519) residuals appear mostly centred around zero, suggesting valid proportional hazards, but are close to the significance threshold. The site had a *p*-value of 0.4305, indicating non-violation of the proportional hazards assumption. There was a noticeable violation of the proportional assumption for the variables tuberculosis, age, viral load, and CD4 count, as their *p*-values were less than 0.05. The analysis suggests that while some covariates may satisfy the proportional hazards assumption, others indicate potential violations. The Stratified Cox model was implemented to address violations of the Cox Proportional Hazards assumption in the next section.

### 3.5 Stratified Cox model

Table 3 presents the results from the Stratified Cox model. The hazard ratio of 0.98 indicates that with each additional year of age, the hazard of CD4 count recovery decreases by 2%. This effect is statistically significant, with a *p*-value of 0.0002, providing strong evidence that age influences CD4 count recovery. As patients age, they may be at increased risk of serious health issues. Moreover, the hazard ratio of 1.47 indicates that higher viral loads markedly increase the risk of poor recovery outcomes. This emphasises the importance of effective viral suppression, which is essential for facilitating CD4 recovery and reducing associated health risks. High viral loads may hinder progress in CD4 counts, undermining the benefits of immune recovery. Additionally, the reduced hazard of 0.60 for females indicates that gender differences play a significant role in treatment outcomes and recovery trajectories.

**Table 3:** Stratified Cox model.

Variable	HR (95% CI)	*p*-value
Age	0.98 (97,0.99)	0.0002
Viral load	1.47 (1.20,1.80)	0.0002
CD4 count	1.0047 (1.0033,1.0060)	<0.0001
Gender	0.60 (0.51,0.71)	<0.0001

### 3.6 Global test

The results in Table 4 suggested proportional hazard assumption violation from both the global test and individual covariates, indicating that the Stratified Cox proportional hazards model may not be appropriate. This is evidenced by *p*-values less than 0.05, and the next section implements the Weibull model as an alternative model.

**Table 4:** Global test.

Variable	Chi-squared	Degrees of freedom	*p*-value
Age	9.74	1	0.0018
Viral load	8.54	1	0.0035
CD4 count	12.21	1	0.00048
Gender	4.63	1	0.0313
Global	31.03	4	<0.0001

### 3.7 Weibull model

Table 5 presents the results from the Weibull Model. Male patients had a significantly lower hazard of CD4 recovery compared with females (HR = 0.589, 95% CI: 0.497–0.698, *p* < 0.001), indicating that males experienced slower immunological recovery. Age was negatively associated with CD4 recovery (HR = 0.984, 95% CI: 0.975–0.993, *p* < 0.001). For each additional year of age, the hazard of recovery decreased, suggesting slower immune reconstitution among older patients. Patients with TB had a significantly reduced hazard of CD4 recovery compared with those without TB (HR = 0.605, 95% CI: 0.489–0.748, *p* < 0.001), indicating that TB co-infection substantially delays immunological recovery. Patients in rural areas had a significantly higher hazard ratio (HR = 1.494, 95% CI: 1.228–1.818, *p* < 0.001), implying rural patients recover faster.

**Table 5:** Weibull model.

Characteristics	Weibull model	
	HR (95% CI)	*p*-value
Gender (Ref: female)*** Male	0.589 (0.497–0.698)	<0.001
Age***	0.984 (0.975–0.993)	<0.001
TB (Ref: no TB present)*** TB	0.605 (0.489–0.748)	<0.001
WHO stage1	0.290 (0.071–1.182)	0.084
WHO stage2	0.264 (0.065–1.072)	0.062
WHO stage3	0.360 (0.089–1.453)	0.151
WHO stage4 (Ref)		
Site (Ref: urban)*** Rural	1.494 (1.228–1.818)	<0.001
Regimen (Ref: first line treatment) Second line treatment	0.974 (0.798–1.189)	0.794
Viral load (Ref: >400copies/mL) ≤400copies/mL***	1.489 (1.213–1.826)	<0.001
Baseline CD4 count***	1.005 (1.004–1.007)	<0.001

***Indicates significant variables.

## 4 Discussion

This study applied time-to-event models to identify factors associated with the timing of CD4 count recovery among people living with HIV who initiated ART with advanced immunosuppression (CD4 < 200 cells/mm^3^), most commonly classified as late presenters. Late presentation has been consistently associated with impaired immune recovery, opportunistic infections, and increased mortality. The high proportion of patients who did not achieve CD4 recovery in our cohort underscores the long-term immunological consequences of delayed entry into care and highlights the importance of early HIV diagnosis and timely ART initiation (25). Previous studies have similarly demonstrated that baseline CD4 count strongly predicts immune reconstitution and that individuals initiating ART at very low CD4 levels may fail to achieve counts exceeding 500 cells/mm^3^ (9, 17).

Several demographic and clinical factors were significantly associated with CD4 recovery, including tuberculosis status, geographic location, viral load, gender, age, and baseline CD4 count. The interpretation of the gender effect on CD4 count recovery indicates a clear disparity in immune reconstitution between males and females. The findings showed that males had a significantly lower hazard of CD4 recovery compared to females (HR = 0.584, *p* < 0.001), indicating slower immunological recovery among men following ART initiation. This result is consistent with previous studies reporting that men experience poorer HIV treatment outcomes, including slower CD4 recovery and higher mortality risk compared to women (26–28). Biological differences in immune function, health-seeking behaviour, later presentation to care, and differences in treatment adherence may contribute to this pattern. Although some evidence suggests that men may demonstrate strong virological responses, women often exhibit stronger early immunological responses to ART. However, this advantage may diminish over time, particularly when accounting for baseline disease severity and other clinical factors (29).

Geographic location was a significant determinant of CD4 count recovery. Patients from rural areas had a significantly higher hazard of CD4 recovery compared to their urban counterparts (HR = 1.307, *p* = 0.002), indicating a faster rate of immunological recovery among rural patients (42). This finding suggests that, within this cohort, rural patients may have benefited from factors such as earlier treatment response, differences in baseline characteristics, or programmatic variations in care delivery. Although prior studies have reported mixed evidence regarding urban–rural disparities in HIV outcomes (30, 31), with many highlighting structural barriers in rural settings (31, 32), our results suggest that rural residence in this context was associated with improved immunological recovery. Differences across studies may reflect variations in healthcare systems, decentralisation of ART services, population characteristics, and baseline disease severity.

Tuberculosis co-infection was associated with reduced CD4 recovery, underscoring the challenge of managing HIV–TB co-morbidity. Patients with prevalent TB were less likely to achieve CD4 counts ≥500 cells/mm^3^ (HR = 0.759, *p* = 0.004), consistent with previous studies reporting impaired immune reconstitution in the presence of TB infection (33–35). TB-related immune activation and systemic inflammation may hinder CD4 restoration despite ART, emphasising the importance of integrated HIV–TB care.

Viral load suppression emerged as a strong predictor of CD4 recovery. Patients with viral loads ≤400 copies/mL experienced significantly faster and more frequent CD4 recovery than those with higher viral loads (HR = 1.467, *p* < 0.001), highlighting the central role of effective virological control in immune reconstitution (36). Although ART adherence was not directly measured in this study, sustained viral suppression likely reflects effective treatment response and continuity of care rather than behavioural factors alone (37, 38). These findings reinforce viral load as a key biological marker linked to immunological recovery.

Age was negatively associated with CD4 recovery, indicating diminished immune reconstitution among older patients (HR = 0.980, *p* < 0.001). This finding is consistent with evidence that immune regenerative capacity declines with age, leading to slower and less complete CD4 recovery following ART initiation (19, 29). Early treatment initiation and closer clinical monitoring may therefore be particularly important in older populations.

In contrast, WHO clinical stage and ART regimen type were not significantly associated with CD4 recovery. The lack of regimen effect may reflect the use of standardised first-line therapies across the cohort, suggesting that baseline clinical and demographic characteristics exert a greater influence on immune recovery than regimen choice alone. This finding supports the continued effectiveness of existing treatment protocols and indicates that switching regimens, when clinically indicated, may not compromise immunological outcomes.

Overall, these findings highlight the multifactorial nature of CD4 recovery following ART initiation. Differences by age, gender, co-infection status, viral suppression, and geographic context suggest that immune reconstitution is shaped by both biological and structural factors. Addressing disparities in access to care, ensuring early diagnosis and treatment, and prioritising sustained viral suppression remain central to improving immunological outcomes in HIV treatment programs.

The introduction of dual-drug regimens in antiretroviral therapy seems to be an effective alternative to conventional three-drug combinations, particularly for treatment-experienced and virologically stable patients (39–41). Advantages of dual drug regimens are reduced toxicity, lower treatment burden, and improved long-term tolerability. However, their effectiveness in individuals presenting with advanced HIV disease (WHO stage 4) remains insufficiently studied. Patients with severe immunosuppression often require robust viral suppression to facilitate optimal immune reconstitution.

### 4.1 Limitations

The findings derived from this study pertain to a specific population in KwaZulu-Natal, which may limit their applicability to other regions or demographics with different healthcare contexts. There may also be biases introduced by the complete case analysis method, which excludes patients with missing data, potentially skewing results if that missing information is related to specific outcomes. We recommend multiple imputation for handling missing data. Addressing adherence as a variable in future studies is essential for understanding its impact on immunological recovery. Additionally, larger, prospective studies could provide more comprehensive insights into the dynamics of CD4 recovery across various populations.

## 5 Conclusion

In conclusion, time-to-event analyses demonstrated significant variability in CD4 recovery following ART initiation. Older age, male sex, urban residence, TB co-infection, and unsuppressed viral load (>400 copies/mL) were independently associated with delayed immune reconstitution. These findings underscore the substantial heterogeneity in immunological response among individuals initiating ART with advanced immunosuppression. Although antiretroviral regimen type was not significantly associated with CD4 recovery in this cohort, delayed or incomplete immune restoration may heighten the risk of HIV-related morbidity and increase the burden on healthcare systems. Targeted interventions and closer monitoring of high-risk subgroups may be warranted to optimise immunologic outcomes and improve long-term prognosis. The identification of baseline clinical and demographic factors associated with slower CD4 recovery may assist in risk stratification and targeted clinical follow-up. Monitoring of viral load levels remains central for assessing virological response to ART, while CD4 count measurements provide complementary information on immunological recovery, particularly among patients initiating treatment with low CD4 counts. While dual antiretroviral therapy (ART) regimens, particularly dolutegravir-based combinations, have demonstrated robust efficacy and durability in maintaining viral suppression across diverse clinical settings, evidence remains limited in individuals presenting late with advanced immunosuppression. Late presenters represent a clinically vulnerable population in whom the dynamics of virologic response, CD4^+^ T-cell recovery, immune reconstitution, and the risk of immune reconstitution inflammatory syndrome may differ from those observed in earlier-stage disease. Future prospective studies and real-world cohort analyses are therefore warranted to specifically evaluate the safety, effectiveness, and immunologic outcomes of two-drug regimens in late presenters, including their impact on long-term immune recovery and clinical endpoints. Such data will be essential to better define the role of dual ART strategies in this high-risk population and to inform individualized treatment approaches.

## Data Availability

Publicly available datasets were analyzed in this study. This data can be found at: South African Centre for the AIDS Program of Research (CAPRISA).
